# Atomic Structure of the Human Sapovirus Capsid Reveals a Unique Capsid Protein Conformation in Caliciviruses

**DOI:** 10.1128/jvi.00298-22

**Published:** 2022-04-18

**Authors:** Naoyuki Miyazaki, Chihong Song, Tomoichiro Oka, Motohiro Miki, Kosuke Murakami, Kenji Iwasaki, Kazuhiko Katayama, Kazuyoshi Murata

**Affiliations:** a Life Science Center for Survival Dynamics, Tsukuba Advanced Research Alliance, University of Tsukuba, Tsukuba, Ibaraki, Japan; b National Institute for Physiological Sciences, Okazaki, Aichi, Japan; c Exploratory Research Center on Life and Living Systems (ExCELLS), National Institutes of Natural Sciences, Okazaki, Aichi, Japan; d Department of Physiological Sciences, School of Life Science, The Graduate University for Advanced Studies (SOKENDAI), Okazaki, Aichi, Japan; e Department of Virology II, National Institute of Infectious Diseases, Tokyo, Japan; f Vaccine and Biomedicine Department, Life Innovation Research Institute, Denka Innovation Center, Denka Co., Ltd., Machida City, Tokyo, Japan; g Laboratory of Viral Infection I, Department of Infection Control and Immunology, Ōmura Satoshi Memorial Institute, Graduate School of Infection Control Sciences, Kitasato University, Tokyo, Japan; University of Kentucky College of Medicine

**Keywords:** sapovirus, *Caliciviridae*, capsid structure, cryo-electron microscopy, single-particle analysis, atomic model

## Abstract

Sapovirus (SaV) is a member of the *Caliciviridae* family, which causes acute gastroenteritis in humans and animals. Human sapoviruses (HuSaVs) are genetically and antigenically diverse, but the lack of a viral replication system and structural information has hampered the development of vaccines and therapeutics. Here, we successfully produced a self-assembled virus-like particle (VLP) from the HuSaV GI.6 VP1 protein, and the first atomic structure was determined using single-particle cryo-electron microscopy (cryo-EM) at a 2.9-Å resolution. The atomic model of the VP1 protein revealed a unique capsid protein conformation in caliciviruses. All N-terminal arms in the A, B, and C subunits interacted with adjacent shell domains after extending through their subunits. The roof of the arched VP1 dimer was formed between the P2 subdomains by the interconnected β strands and loops, and its buried surface was minimized compared to those of other caliciviruses. Four hypervariable regions that are potentially involved in the antigenic diversity of SaV formed extensive clusters on top of the P domain. Potential receptor binding regions implied by tissue culture mutants of porcine SaV were also located near these hypervariable clusters. Conserved sequence motifs of the VP1 protein, “PPG” and “GWS,” may stabilize the inner capsid shell and the outer protruding domain, respectively. These findings will provide the structural basis for the medical treatment of HuSaV infections and facilitate the development of vaccines, antivirals, and diagnostic systems.

**IMPORTANCE** SaV and norovirus, belonging to the *Caliciviridae* family, are common causes of acute gastroenteritis in humans and animals. SaV and norovirus infections are public health problems in all age groups, which occur explosively and sporadically worldwide. HuSaV is genetically and antigenically diverse and is currently classified into 4 genogroups consisting of 18 genotypes based on the sequence similarity of the VP1 proteins. Despite these detailed genetic analyses, the lack of structural information on viral capsids has become a problem for the development of vaccines or antiviral drugs. The 2.9-Å atomic model of the HuSaV GI.6 VLP presented here not only revealed the location of the amino acid residues involved in immune responses and potential receptor binding sites but also provided essential information for the design of stable constructs needed for the development of vaccines and antivirals.

## INTRODUCTION

**FIG 1:**
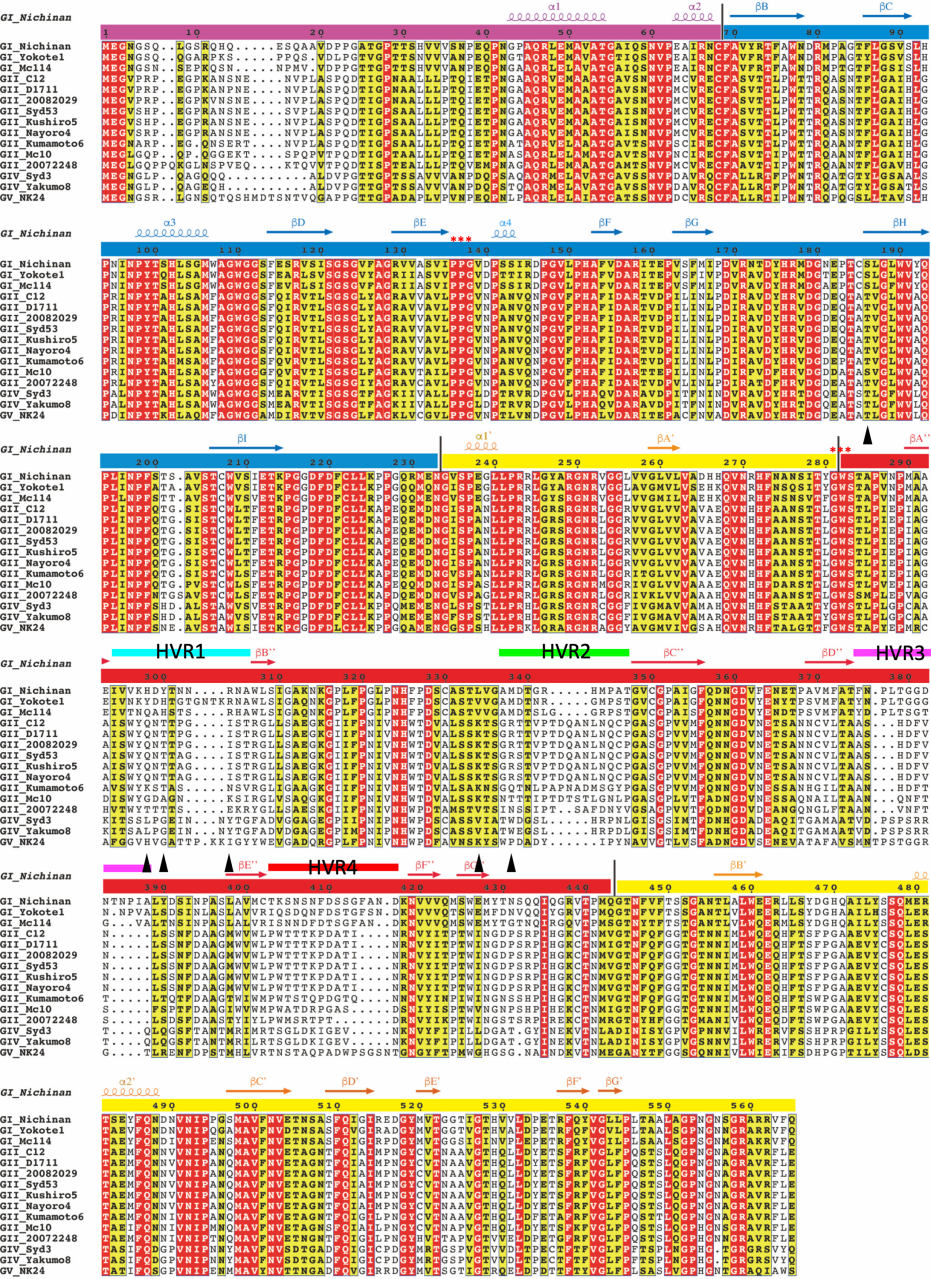
Sequence alignment of the amino acids of the representative HuSaV VP1 proteins. The N-terminal arm (NTA), S domain, and P1 and P2 subdomains are indicated by colored bars (purple, NTA; blue, S; yellow, P1; red, P2). Secondary structural elements are shown above the sequences as a spiral (α-helix) or an arrow (β-sheet), having the same color as those of similar domain regions. Letters on red and yellow backgrounds indicate identical and similar amino acids, respectively, based on the Risler matrix (40). Sequence gaps are shown as dotted lines. The four hypervariable regions are indicated by bars of different colors and labeled HVR1 through HVR4, suggesting that they are common epitopes on these HuSaVs. The well-conserved motifs PPG and GWS of caliciviruses are indicated by asterisks. The figure was drawn using ESPript (43). Sequence data were provided by GenBank (Table 1).

Sapovirus (SaV) belongs to the genus *Sapovirus* (species *Sapporo virus*) of the family *Caliciviridae*, which causes acute gastroenteritis in humans and animals worldwide (1). Outbreaks primarily occur in semienclosed spaces. Especially, outbreaks caused by foodborne infections have commonly been reported. SaV was first discovered in 1976 by electron microscopy (EM) of human diarrhea samples (2). To date, SaVs have been identified with greater genetic and antigenic diversities, which are classified into 19 genogroups based on the VP1 (viral protein 1) sequence (genogroup I [GI] to GXIX) (3). Of these strains, four genogroups, GI, GII, GIV, and GV, infect humans, while the remaining genogroups infect animals: pigs (GIII and GV to GXI), sea lions (GV), minks (GXII), dogs (GXIII), bats (GXIV and GXVI to GXIX), and rats (GXV). Human sapoviruses (HuSaVs) have been further subdivided into 18 genotypes (GI.1 to GI.7, GII.1 to GII.8, GIV.1, and GV.1 to GV.2). Representative strains with their amino acid sequences are shown in Fig. 1. The sequence data were provided by GenBank (Table 1).

**TABLE 1:** Fifteen sapovirus strains

Strain	Genogroup	Genotype	GenBank accession no.
Mc114	GI	1	AY237422
Yokote1	GI	5	AB253740
Nichinan	GI	6	AB455803
Mc10	GII	2	AY237420
C12	GII	3	AY603425
20082029	GII	3	AB630068
D1711	GII	3	AB522391
Syd53	GII	3	DQ104360
Kushiro5	GII	3	AB455793
Nayoro4	GII	3	AB455794
Kumamoto6	GII	4	AB429084
20072248	GII	7	AB630067
Syd3	GIV	1	DQ104357
Yakumo8	GIV	1	AB455795
NognKhai24 (NK24)	GV	1	AY646856

HuSaV contains a positive-sense single-stranded RNA genome of approximately 7.5 kb in length (4). The viral genome consists of two or three open reading frames (ORFs) (ORF1 to ORF3), depending on the genogroup. ORF1 encodes a polyprotein that undergoes proteolytic cleavage to form nonstructural proteins (NS1 to NS7) and a major capsid protein (VP1). ORF2 encodes a minor capsid structural protein (VP2), and ORF3 encodes nonstructural proteins, but their actual functions are not clear (5–7). The VP1 protein is solely responsible for most capsid-related processes such as assembly, host interactions, and immunogenicity. However, until the recent establishment of the SaV cultivation system (8), the lack of a sufficient viral replication system, such as the actual target cells in the host, has hampered our understanding of the SaV replication strategy, etiology, and immunogenicity.

The *Caliciviridae* family is currently classified into 11 established genera: *Bavovirus*, *Lagovirus*, *Minovirus*, *Nacovirus*, *Nebovirus*, *Norovirus*, *Recovirus*, *Salovirus*, *Sapovirus*, *Valovirus*, and *Vesivirus* (9). The atomic structures of calicivirus VLPs (virus-like particles) or virions have been determined only in viruses of three established genera, norovirus (10–13), lagovirus (14), and vesivirus (15–17), using X-ray crystallography and cryo-electron microscopy (cryo-EM). For SaV, the structure has been reported at only low to intermediate resolution (18, 19). Among these, calicivirus virions have a common capsid shell structure consisting of 180 copies of VP1 arranged in T=3 icosahedral symmetry. According to their positions, the VP1 proteins are also designated the A, B, and C subunits in the icosahedral asymmetric unit, forming quasiequivalent A/B dimers and equivalent C/C dimers (10). Moreover, each VP1 capsid monomer contains two principal domains, the shell (S) domain and the protruding (P) domain, in addition to an N-terminal arm (NTA) interconnecting the S domains in the capsid shell.

The S domain represents the most conserved region of the amino acid sequence among caliciviruses, consisting of a classical eight-stranded β sandwich commonly found in T=3 and P=3 icosahedral viruses (20). The fundamental function of the S domain is to form a continuous icosahedral inner capsid shell responsible for protecting the viral genome from the outer environment. In contrast, the structure and amino acid sequence of the P domain vary considerably among caliciviruses because this domain is primarily involved in virus-host interactions and immunogenicity (19). P-domain dimers form protrusions on the inner capsid shell composed of S-domain dimers. Each P domain can also be further divided into two subdomains called P1 and P2, forming the upper and lower parts of the P domain, respectively (12). Although little sequence similarity exists, protein folds in the P1 and P2 subdomains are conserved among caliciviruses (19). Relative interactions and conformations between the P domains of the VP1 dimer are also conserved within the genus, rarely extending between genera. For example, while the P2 subdomain is primarily involved in dimeric interactions in vesiviruses and lagoviruses, the P1 and P2 domains closely participate in dimeric interactions in noroviruses (12, 14, 17). SaV has a P-domain dimer structure similar to that of the former group because it shows a typical arched protruding dimer connected at the top of the P domain (18). Therefore, to understand the immunogenicity and interaction between the virus and the host, based on the intergenus diversity of caliciviruses, it is desirable to elucidate the atomic structures of other caliciviruses, including SaV.

Here, we successfully produced a HuSaV GI.6 VLP and determined its capsid structure at a 2.9-Å resolution using single-particle cryo-EM. The cryo-EM map was used to build the first atomic model of the SaV capsid. The atomic model revealed a unique capsid protein conformation in caliciviruses. All NTAs in the A, B, and C subunits interacted with adjacent shell domains after extending through their subunits. The roof of the arched VP1 dimer was formed with the interconnected β strands and loops, exhibiting the smallest buried surface between the P domains compared to other caliciviruses. The results provide a structural basis for developing therapeutic agents effective in treating HuSaV infectious disease.

## RESULTS

### 3D reconstruction of the HuSaV GI.6 VLP.

**FIG 2:**
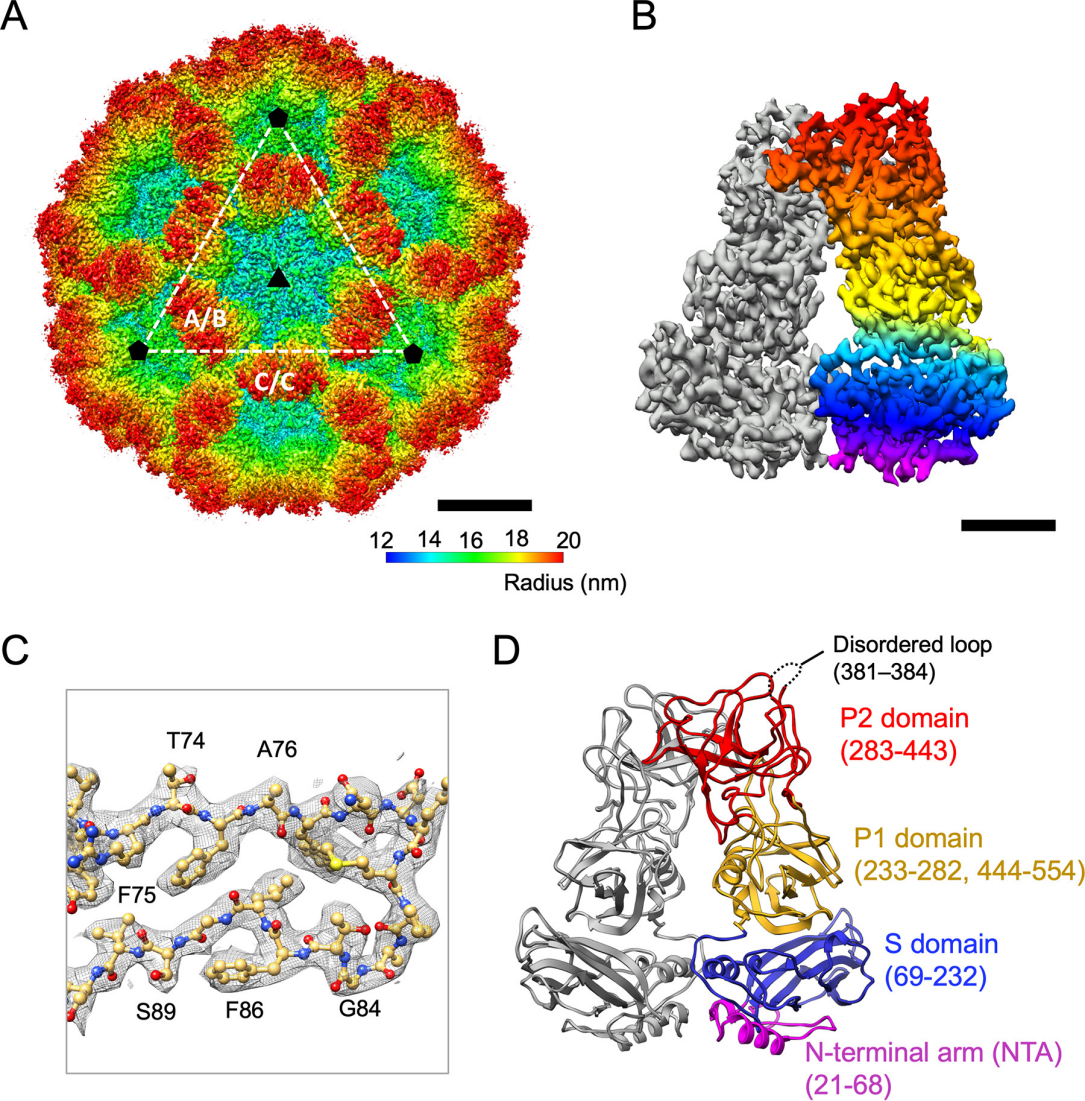
Cryo-EM map of the HuSaV GI.6 VLP and atomic structure of the VP1 protein. (A) Surface representation of the 2.9-Å-resolution cryo-EM map of the HuSaV GI.6 VLP. The map is colored according to the radius of the particle. Icosahedral 3- and 5-fold axes are indicated with black triangles and black pentagons, respectively. Icosahedral 2-fold axes are placed in the middle of the dotted white lines. The A/B and C/C VP1 dimers are also labeled. Bar, 10 nm. (B) Surface representation of the cryo-EM map of a VP1 C/C dimer. The VP1 monomer is in gray. The NTA and S domains are in magenta and blue, and the P1 and P2 subdomains are in yellow and red, respectively. Bar, 2 nm. (C) A cryo-EM density map and the corresponding atomic model of the HuSaV GI.6 VP1 protein are shown as gray mesh and ball-and-stick representations, respectively. Oxygen, nitrogen, carbon, and sulfur atoms are in red, blue, gold, and yellow, respectively. (D) Ribbon diagram of the HuSaV C/C dimer. The NTA (residues 21 to 68), the S domain (residues 69 to 232), and the P1 (residues 232 to 282 and 444 to 554) and P2 (residues 283 to 443) subdomains in the monomer are colored as described above for panel B. The disordered loop (residues 381 to 384) on the capsid surface is indicated by a dotted line.

The capsid protein VP1 of HuSaV from the Nichinan strain in genogroup I (GI.6) (21) was expressed in a baculovirus expression system, after which VLPs self-assembled and secreted from these cells were collected from the culture medium and purified. Next, a three-dimensional (3D) structure was reconstructed from VLPs at a 2.9-Å resolution using cryo-EM single-particle analysis (Fig. 2). The cryo-EM map clearly showed a T=3 icosahedral symmetry with 90 protrusions consisting of 180 copies of the VP1 protein and forming icosahedral 2-fold axes (C/C dimers) and quasi-2-fold axes (A/B dimers) (Fig. 2A). The VP1 dimer showed an arched protruding domain on the S domains, connected at the top part of the P2 subdomains (Fig. 2B). The bulky side chains were also clearly resolved in the cryo-EM map, after which the atomic models were unambiguously built for the VP1 protein (Fig. 2C).

### Structures of the VP1 proteins of SaVs.

The cryo-EM map allowed atomic modeling of residues 38 to 554 for subunit A, 21 to 554 for subunit B, and 21 to 554 for subunit C, except for one disordered loop (residues 381 to 384) for all subunits (Fig. 2D). The overall structure of the capsid VP1 protein of HuSaV GI.6 comprises two principal domains, the S (residues 69 to 232) and P (residues 233 to 554) domains, in addition to an NTA (residues 21 to 68 in subunits B and C and residues 38 to 68 in subunit A) run inside the capsid shell. The P domain was further divided into two subdomains, P1 (residues 233 to 281 and 444 to 554) and P2 (residues 282 to 443). These domain boundaries were in close agreement with previous results based on the homology model constructed on an 8.5-Å-resolution cryo-EM map of the HuSaV chimeric VLP (Yokote/Mc114) (18). A disordered region (residues 381 to 384) located on the exterior surface of the viral particle was placed in the loop between βD″ and βE″ of the P2 subdomain (dashed circles in Fig. 2D). This region was included in one of the most variable amino acid sequences in HuSaVs (22), called hypervariable region 3 (HVR3) (Fig. 1).

### Structures of the SaV shell domains.

**FIG 3:**
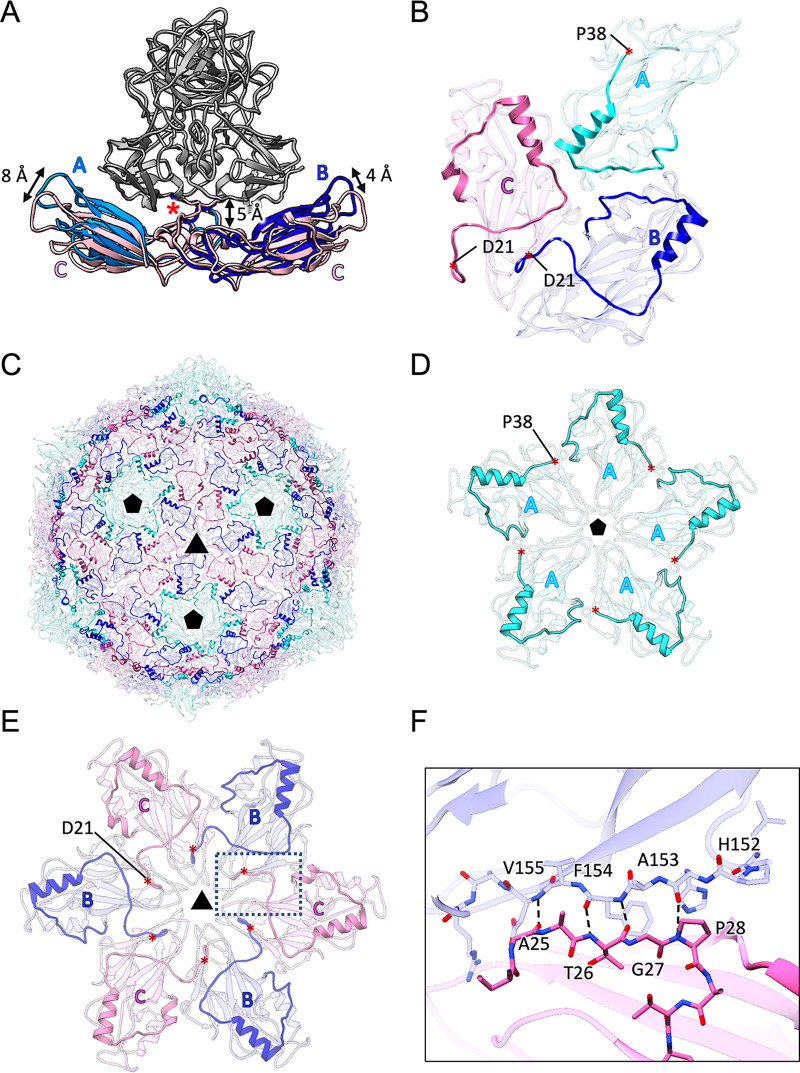
Structures of the shell domains of SaV. (A) Ribbon diagrams of the SaV A/B and C/C dimers in the S domain in light blue/dark blue and pink, respectively. The icosahedrally independent A/B and C/C dimers showed different conformations between the two S domains. These are flat in the C/C dimer but inwardly bent at 13° in the A/B dimer, causing an 8-Å raise at the A-monomer end and a 4-Å rise at the B-monomer end. Red asterisks show the loops connecting the S and P domains undergoing a hinge-like motion to adapt to the two conformers. (B) Ribbon diagram of the S domains of the A, B, and C subunits viewed from inside the capsid. NTAs of the A, B, and C subunits interact with adjacent subunits after extending through their subunits. Residues 22 to 37 of the NTA in the A subunit were disordered. (C) NTAs of the A, B, and C monomers on the S-domain capsid are highlighted using colors similar to those in panel B. Black triangles and black pentagons indicate the 3- and 5-fold axes of the T=3 asymmetric units. (D) NTA network of the VP1 proteins on the 5-fold axis. Shown is a ribbon diagram of the S domains around an icosahedral 5-fold axis, viewed from inside the capsid. NTAs of the A subunits are highlighted in cyan (residues 38 to 68). (E) NTA network of the VP1 protein on the 3-fold axis. Shown is a ribbon diagram of S domains around an icosahedral 3-fold axis viewed from inside the capsid. NTAs of the B and C subunits are highlighted in blue (residues 21 to 68) and pink (residues 21 to 68). (F) Close-up view of the dotted box in panel E. An intersubunit β-sheet was formed between the adjacent S domains by the NTA from the B and C subunits on the 3-fold axis.

The icosahedrally independent A/B and C/C dimers showed different conformations primarily between the S domains of each dimer, as reported previously for other T=3 viruses (23) (Fig. 3A). While the two S domains of the C/C dimer were interconnected in a flat conformation, those in the A/B dimer were interconnected in a 13° inward-bend conformation. The interconnection caused an 8-Å raise at the S-domain end of the A monomer and a 4-Å raise at the S-domain end of the B monomer (Fig. 3A). The loop connecting the S and P domains in a β-turn (residues 231 to 234) underwent a hinge-like motion to adapt to the two conformers (red asterisks in Fig. 3A). The P-domain dimers were elevated ∼5 Å from the S domain (Fig. 3A), as previously reported for the HuSaV chimeric VLP cryo-EM map (18), which functioned to avoid a clash between the S and P domains in the bending conformation of the A/B dimer.

As a novel structure of the calicivirus capsid, all NTAs from the A, B, and C subunits interacted with adjacent shell domains after extending through their subunits (Fig. 3B). This is different from those of other caliciviruses previously reported (see Discussion for details). NTAs with residues 21 to 37 in the B and C subunits interacted with adjacent subunits around the icosahedral 3-fold axes (blue and red in Fig. 3C and E), although the NTA was disordered in the A subunit, and no NTA network was identified around the 5-fold axes (Fig. 3D). Amino acid residues A25 to P28 in the NTAs of B and C subunits formed a short intersubunit β-sheet with amino acid residues H152 to V155 in the adjacent subunit (Fig. 3F). Therefore, the NTA network around the 3-fold axes stabilized the pseudohexameric units in the SaV capsid, although the NTA network around the 5-fold axes is presently unclear.

### The function of conserved motifs in SaV VP1 protein.

**FIG 4:**
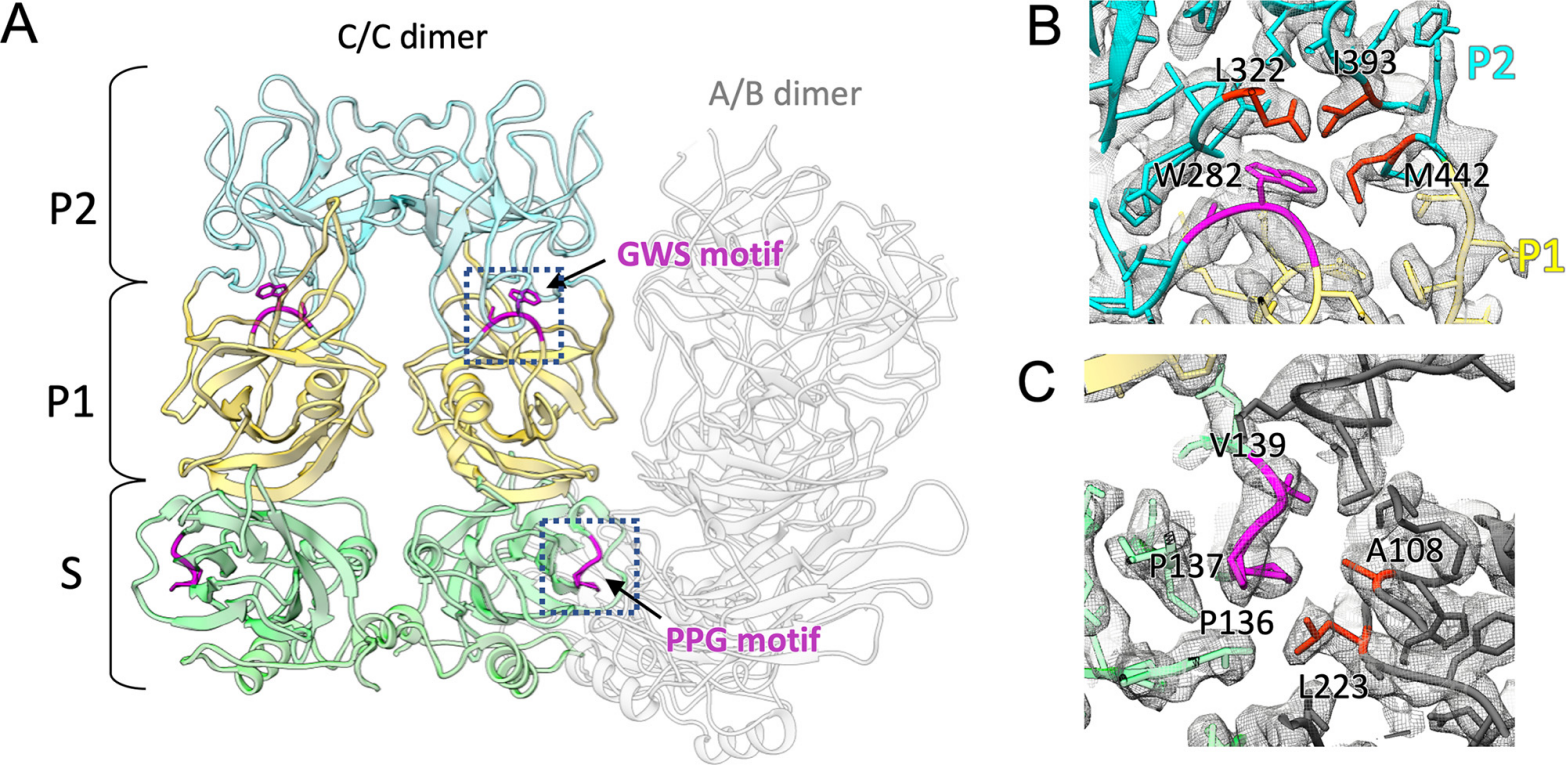
Conserved motifs of the calicivirus VP1 proteins. (A) Two well-conserved motifs in the calicivirus VP1 proteins, “PPG” and “GWS,” were found at residues 136 to 138 and 281 to 283 of HuSaV GI.6. The PPG motif was located in the intersubunit interface between the S-domain dimers, while the GWS motif was located at the boundary between the P1 and P2 subdomains. (B) The GWS motif in the P1 subdomain interacted with L322, I393, and M442 of the P2 subdomain. (C) The PPG motif forming hydrophobic interactions with A108 and L223 of the adjacent S domain.

A previous study identified two well-conserved motifs of the calicivirus VP1 protein, “PPG” and “GWS,” at residues 136 to 138 and 281 to 283 of HuSaV GI.6, respectively (1) (asterisks in Fig. 1). In our study, the PPG motif formed a β-turn consisting of “PPGV” residues between βE and α4 in the S domain (Fig. 4A). We observed that the motif was not exposed to the viral surface, but it was located at the interdomain interface between the S-domain dimers. This observation suggests that the conserved PPG motif was involved in stabilizing the viral shell through hydrophobic interactions with A108 and L223 in the adjacent S-domain dimer (Fig. 4C). Alternatively, the GWS motif was located at the boundary between the P1 and P2 subdomains (Fig. 4A). The aromatic side chain of W282 in the motif was inserted from the P1 subdomain into the P2 subdomain, forming a hydrophobic core with L322, I393, and M442 in the P2 subdomains (Fig. 4B). The GWS motif in the loop between βA′ and βA″ was also not exposed to the viral surface like the motif PPG, suggesting that this conserved motif was potentially involved in stabilizing P-domain formation in the SaV capsid.

### Interactions between P domains in the dimeric protrusion.

**FIG 5:**
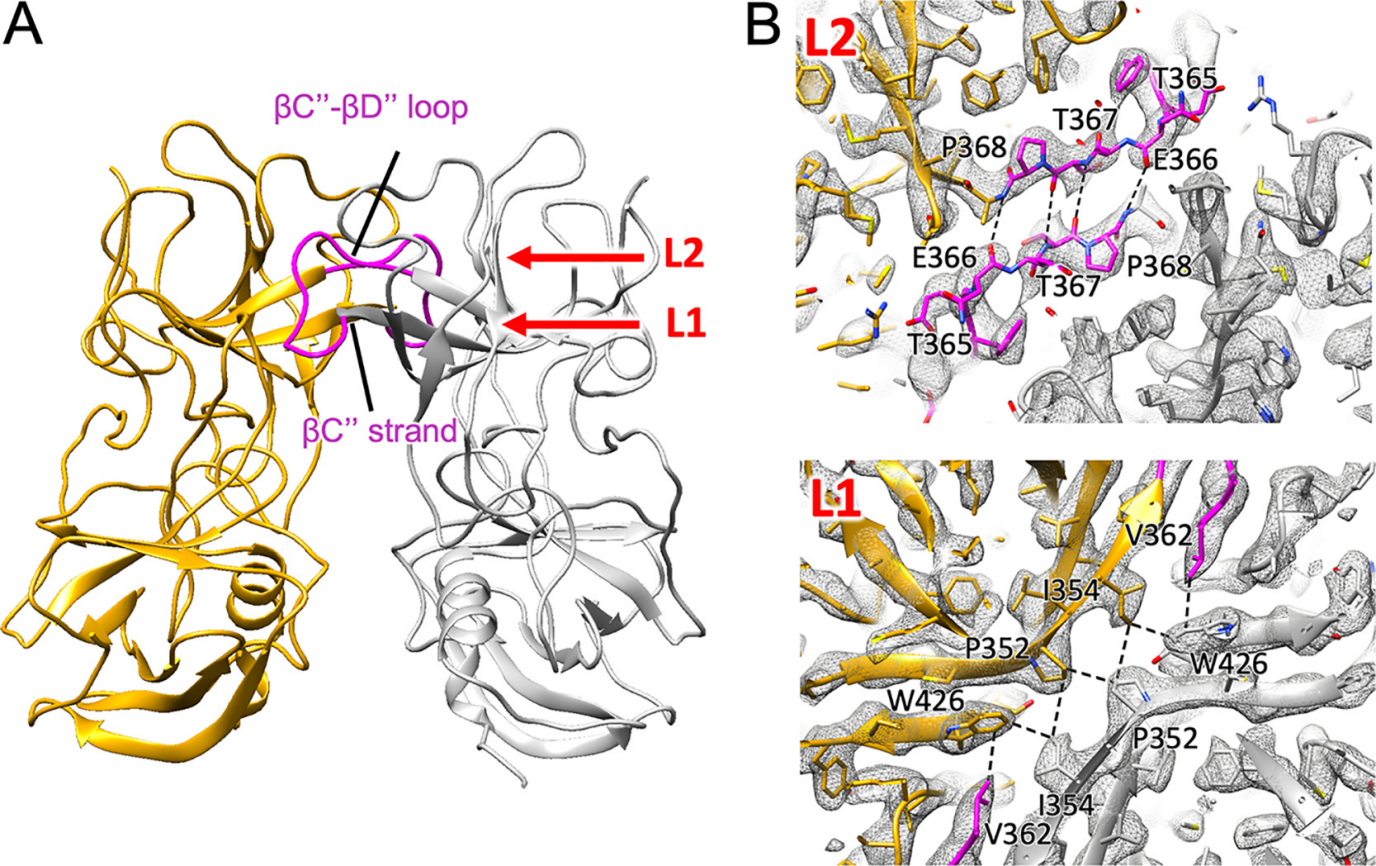
Interactions between two P domains in the SaV VP1 dimer. (A) Dimeric interactions in the P domains occurred between two P2 subdomains. The βC″ strand (residues 348 to 356) and the βC″-βD″ loop (residues 357 to 368) interacted with their counterparts in the paired VP1 proteins (L1 and L2), respectively, forming the double-layer roof of the arched protruding dimer. (B) At layer L1, a pair of βC″ strands running antiparallel interacted with each other in several hydrophobic interactions. At layer L2, a pair of βC″-βD″ loops running antiparallel also interacted with each other in several hydrophobic interactions.

Interactions within the SaV VP1 dimer occurred in the outermost region of the P domain, except for the inner S domain, forming an arched protruding dimer (Fig. 5A) similar to those of lagovirus and vesivirus (18). However, the high-resolution structure at a 2.9-Å resolution revealed that the amino acid residues involved in the interactions between the P domains created a unique dimeric conformation. In the HuSaV VP1 dimer, the βC″ strand (residues 348 to 356) and the following βC″-βD″ loop (residues 357 to 368) interacted with the counterparts in the paired VP1 proteins, forming a double-layer roof at the arched protruding dimer (L1 and L2 in Fig. 5). In layer L1, a pair of the βC″ strands running antiparallelly interacted with each other in several hydrophobic interactions (L1 in Fig. 5B). In layer L2, a pair of the βC″-βD″ loops running antiparallelly also interacted with each other in several hydrophobic interactions (L2 in Fig. 5B). Due to these interactions, it forms a unique arched protruding VP1 dimer of SaV with a thin roof.

### Localization of amino acids involved in the immunological diversity of HuSaVs.

**FIG 6:**
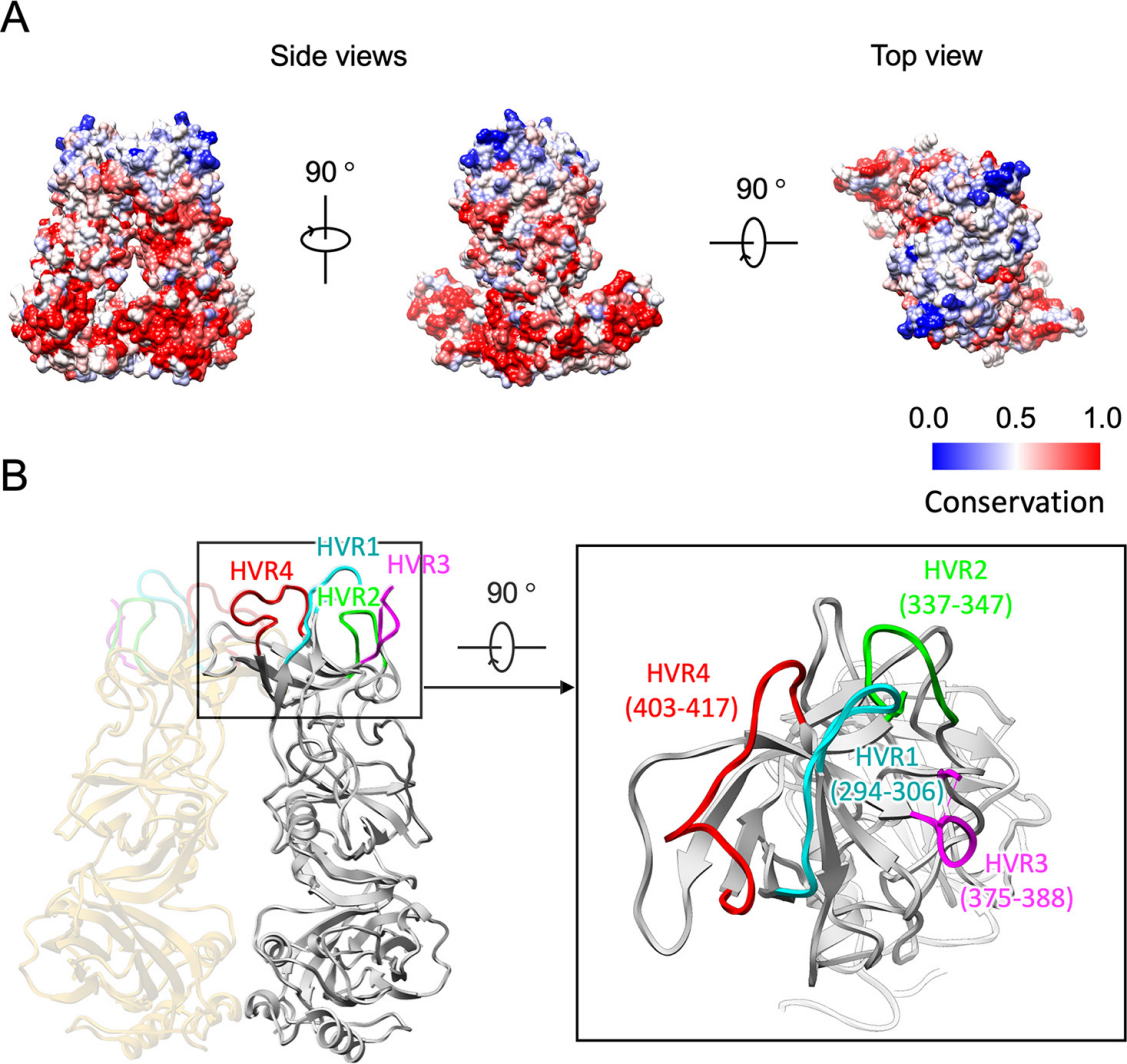
Localization of amino acids involved in the antigenic diversity and immunogenicity of HuSaV. (A) Conservation of amino acid residues of the HuSaV VP1 proteins mapped onto the molecular surface of the VP1 dimer. Red indicates the most conserved area of HuSaV, and blue indicates the least conserved area of HuSaV. These areas are listed in Fig. 1. (B) Hypervariable regions (HVRs) of the P domain in HuSaVs. Four HVRs, HVR1 to HVR4, in cyan, green, magenta, and red, respectively, form extensive clusters on top of the P domain.

To examine the antigenic diversity and immunogenicity of HuSaVs, the amino acid conservation across human GI, GII, GIV, and GV SaVs listed in Fig. 1 was mapped to the 3D structure of the VP1 dimer (Fig. 6A). The amino acid residues in the P2 subdomain on the viral surface had extremely diverged, whereas those in the S domain and the P1 subdomains were highly or intermediately conserved. Moreover, primary sequence comparison showed various insertions and deletions among HuSaV strains in the P2 subdomains (Fig. 1). Residues 294 to 306 between βA″ and βB″, residues 337 to 347 between βB″ and βC″, residues 375 to 388 between βD″ and βE″ (including a disordered region from residues 381 to 384), and residues 403 to 417 between βE″ and βF″ showed significant sequence variabilities among the HuSaVs, which were designated hypervariable regions 1 to 4 (labeled HVR1, HVR2, HVR3, and HVR4 in Fig. 1). The antigenic diversity and immunogenicity of HuSaVs were primarily produced in these four hypervariable regions (22). The atomic model showed that these regions form extensive clusters at the top of the P domain, similar to those of other caliciviruses (19), but they were more extensive than those of the other viruses (Fig. 6B). These large hypervariable clusters suggested that SaV had acquired a great variety of antigenicity in humans and animals. In contrast, the loops and strands in the P2 subdomain, which are not exposed to the outer surface and are possibly involved in dimeric interactions, are highly conserved (areas other than HVR1 to -4 in the P2 subdomain in Fig. 1).

### Localization of amino acids involved in receptor binding in HuSaV.

**FIG 7:**
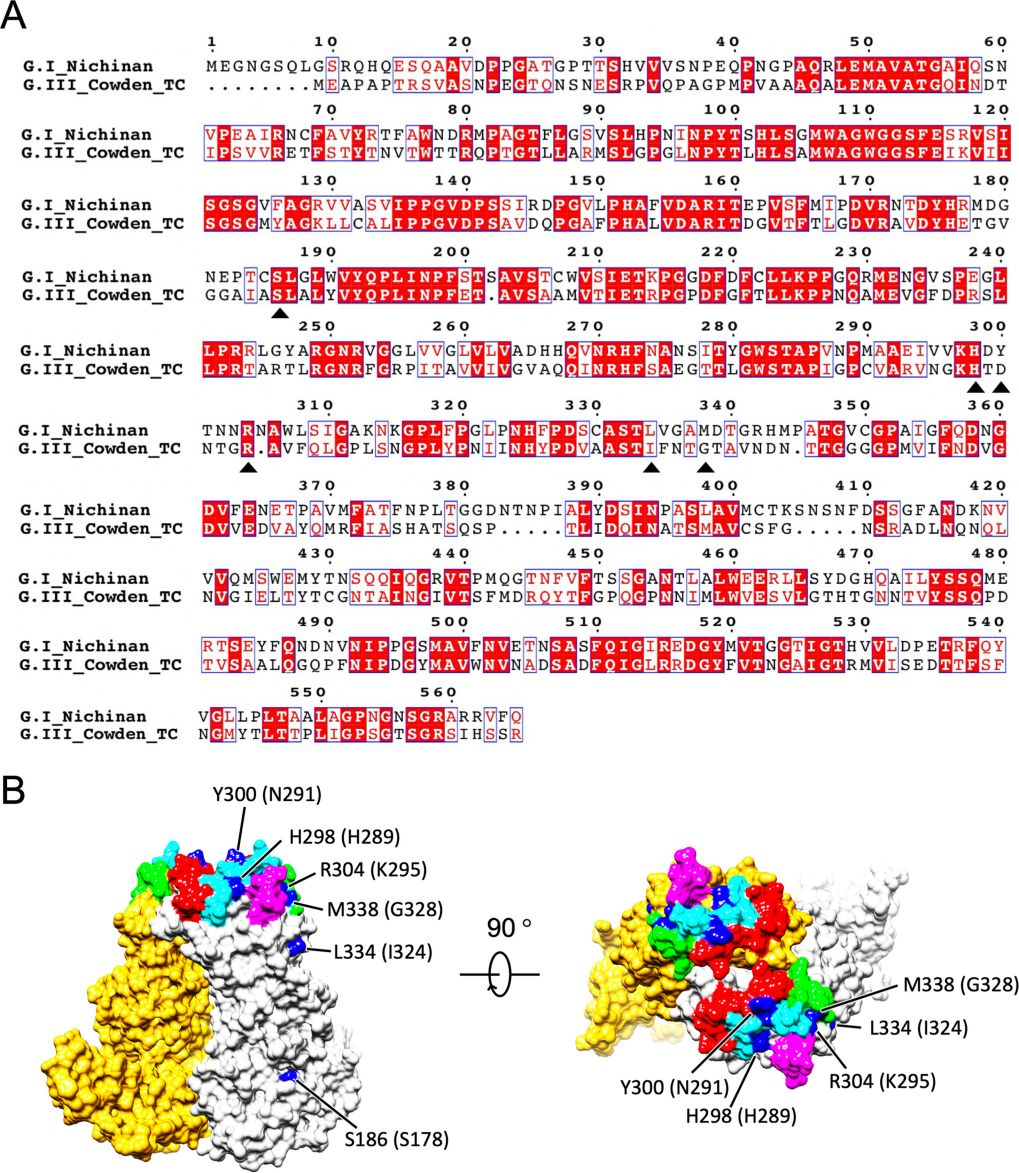
Possible receptor binding positions in HuSaV. (A) Amino acid sequence of HuSaV GI.6 (Nichinan strain) compared with that of the tissue-culture-adapted (TC) PoSaV GIII Cowden strain. The 6 amino acid substitutions in the TC mutant are indicated by black arrowheads. (B) The corresponding amino acid residues for the 6 mutation positions in the TC-type PoSaV Cowden strain were mapped onto a molecular surface of the HuSaV VP1 dimer. The four essential (residues S178, H289, I324, and G328) and two functional (residues N291 and K295) mutation positions are labeled and shown in blue. Amino acid residues in the TC-type PoSaV Cowden strain are enclosed in parentheses. HVR1 to -4 are further marked and shown in cyan, light green, magenta, and red, respectively.

Cell receptor molecules for HuSaV have not been identified so far. Therefore, the host recognition mechanism of HuSaV remains unknown. The porcine sapovirus (PoSaV) Cowden strain in GIII was the only culturable sapovirus until recently, showing mutations adapted to tissue culture (24). Compared to the wild-type (WT) PoSaV Cowden strain, tissue-culture-adapted (TC) PoSaV had 6 amino acid substitutions in its capsid protein (24). Four of the six amino acid substitutions in VP1 (residues C178S, Y289H, M324I, and E328G) were critical for cell culture adaptation of the PoSaV Cowden strain. For the other two substitutions in VP1 (residues D291N and R295K), the revertants enhanced viral replication *in vivo* and induced higher levels of serum and mucosal antibody responses (24). We mapped these four essential and two functional mutations to the primary sequence of HuSaV (Fig. 7A). The corresponding residues of HuSaV for the six TC mutations were S186, H298, Y300, R304, L334, and M338 (black arrowheads in Fig. 7A), and these residues were mapped onto the molecular surface of the HuSaV VP1 dimer (blue in Fig. 7B). Except for one residue, S186, in the S domain, the other five residues in the P domain were exposed to the external environment and located on the receptor-accessible surface, suggesting their essential involvement in the P domain for receptor binding in PoSaV (25). The HuSaV receptor molecule can differ from the PoSaV receptor molecule because the 6 residues were unconserved in HuSaV (black arrows in Fig. 7A), but the position of the receptor binding site will not be significantly different from these areas. Interestingly, the potential receptor binding sites were further located near these HVRs on the P domain of HuSaV (Fig. 7B). These observations are understandable because both of these residues must be located at the virus-cell interface, but further investigation will reveal the relationship between hypervariable regions and receptor binding sites.

## DISCUSSION

Here, we successfully generated a VLP of a HuSaV GI.6 strain using a baculovirus expression system, and the atomic structure of the VP1 capsid was determined at a 2.9-Å resolution by single-particle cryo-EM. The structure revealed the unique architecture of the VP1 protein in caliciviruses and provided valuable information on the vast variety of HuSaV genetics and antigenicities.

In caliciviruses, the S domains of the VP1 protein were interconnected to each other. They form a stable inner capsid shell in which the NTAs extending from the A, B, and C monomers form various interactions with the adjacent S domains at icosahedral 2-, 3-, and 5-fold axes. We had previously reported that the NTA conformation of caliciviruses is structurally divided into two groups (11). One group, primarily to which norovirus belonged (10–13), shows that the NTAs run along the bottom edge of the S domain, after which the terminal residues interact with the adjacent S domain. Another group, to which vesivirus belonged (15–17), shows that the NTAs run along the bottom edge of the adjacent S domain, after which the terminal residues come across the bottom surface of the adjacent S domain along the way and finally interact with the further adjacent S domain. Interestingly, the atomic model of SaV showed that the NTA conformation had characteristics of both groups. NTAs ran along the bottom edge of the S domain, but the terminal residues of the B and C monomers came across the bottom surface of the S domain along the way, finally interacting with the adjacent S domain (Fig. 3B). The NTAs of the A monomer also showed a similar conformation, but the terminal structure was unclear because it was disordered on the way (Fig. 3D). The NTA conformation of lagovirus was also proposed to be similar to that of SaV, but this is presently unclear because all NTA ends are disordered along the way (14). These observations showed that SaV VP1 had a unique structure in its shell domain.

**FIG 8:**
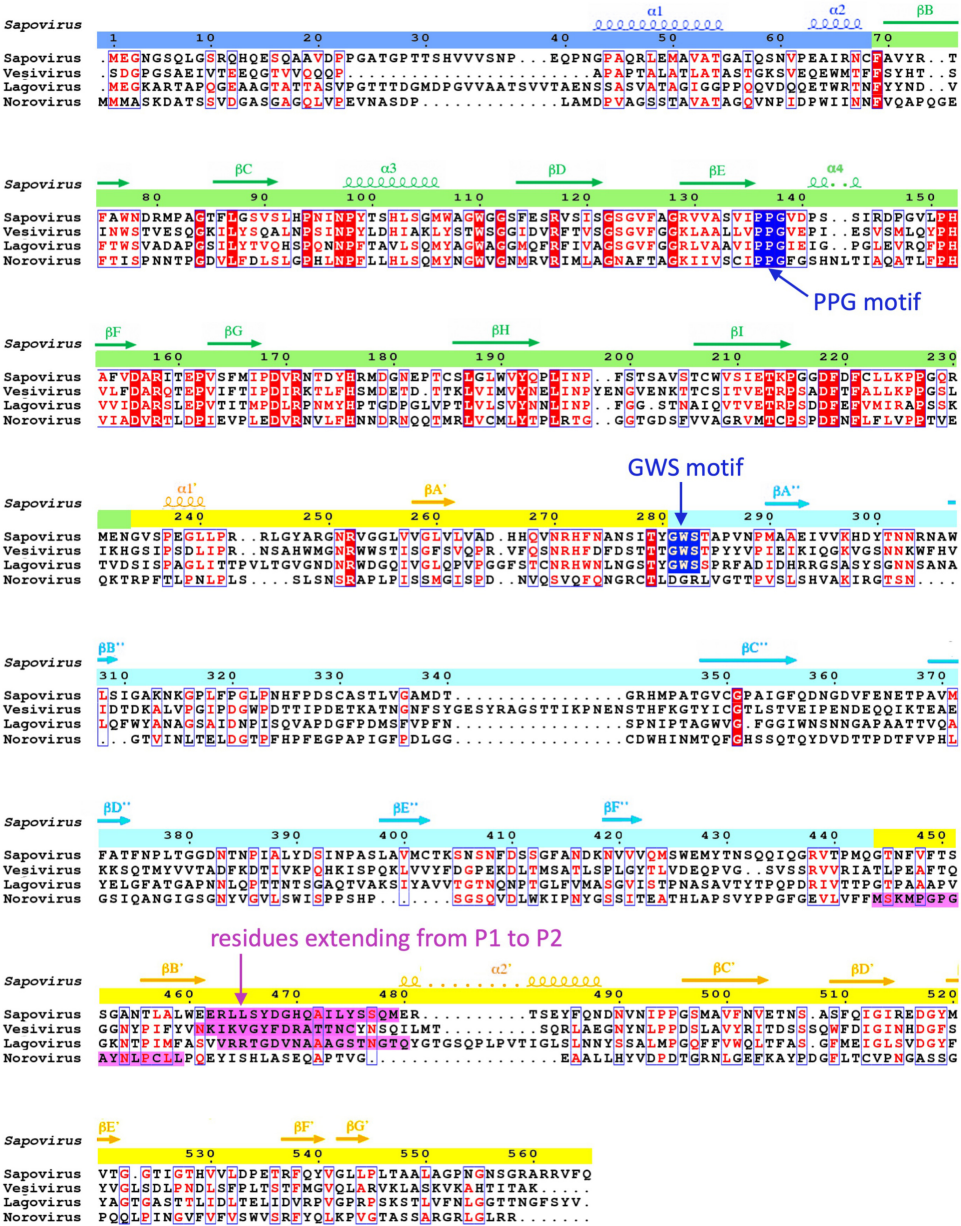
Amino acid sequence comparison of four caliciviruses whose atomic structures have been reported. The amino acid sequence of sapovirus GI.6 (Nichinan strain) was compared with those of vesivirus (PDB accession number 2GH8), lagovirus (PDB accession number 3J1P), and norovirus (PDB accession number 1IHM). The major PPG and GWS conserved motifs in calicivirus VP1 proteins are shown in blue. The PPG motif was preserved in VP1s of sapovirus, lagovirus, vesivirus, and norovirus, while the GWS motif was conserved in VP1s of these viruses, except for norovirus. The residues extending from the P1 to the P2 subdomains are shown in magenta. The figure was drawn by ESPript (43).

**FIG 9:**
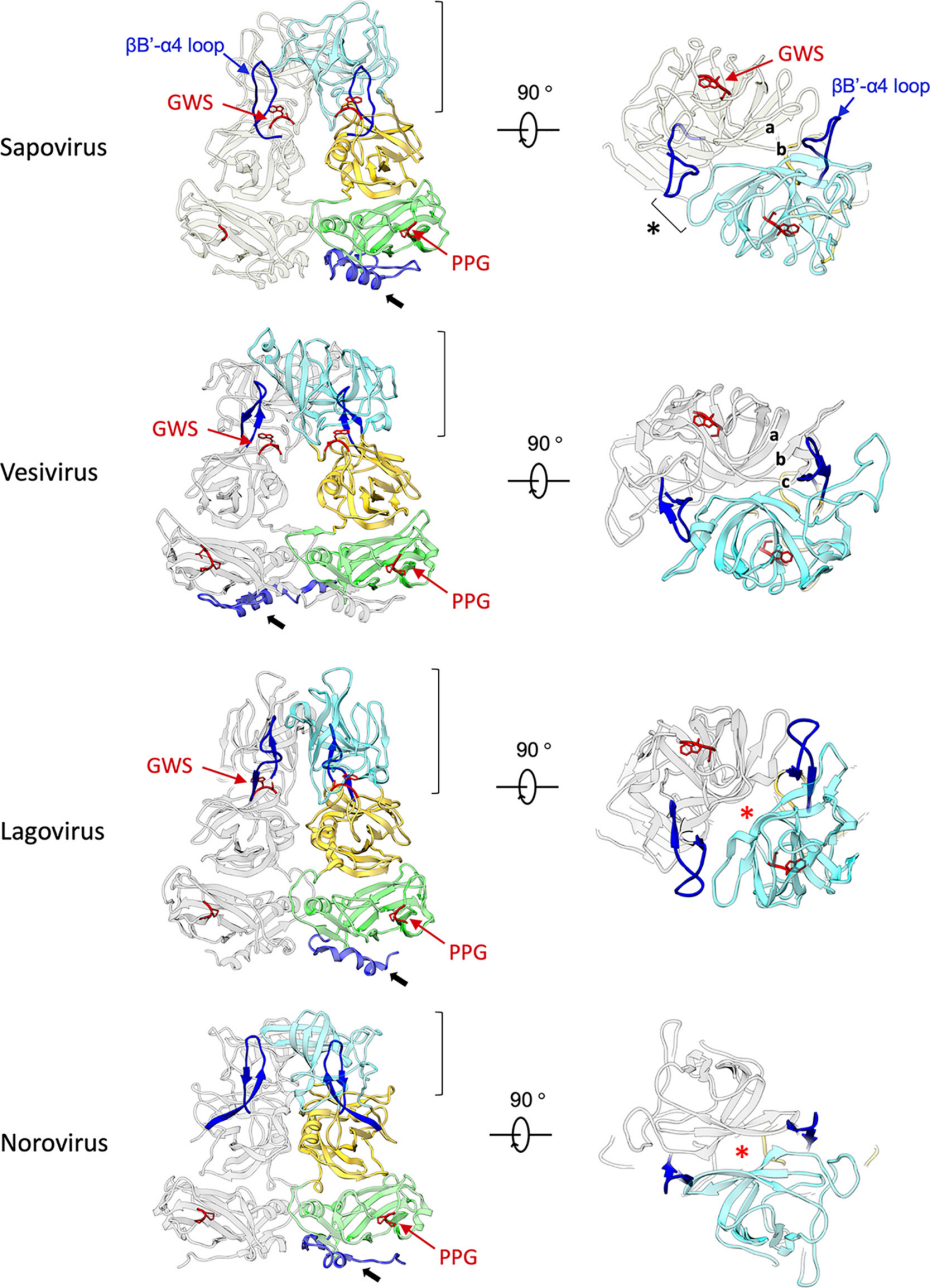
Structural comparison of the VP1 dimers in four caliciviruses whose atomic structures have been reported. Ribbon diagrams of the VP1 dimers of sapovirus GI.6 (PDB accession number 7DOD), vesivirus (PDB accession number 2GH8), lagovirus (PDB accession number 3J1P), and norovirus (PDB accession number 1IHM) are presented. The P2 subdomains viewed from the external side are in the right panels. The major conserved motifs PPG and GWS are indicated and labeled in red. The residues extending from the P1 to the P2 subdomains, shown in dark blue, stabilize the VP1 dimer by interacting with the loop in the adjacent P2 subdomain (black asterisk). NTAs are labeled blue and specified with black arrows. In sapovirus, the roof of the arched VP1 dimer was formed through interactions between antiparallel β-strands (a) and between antiparallel loops (b), exhibiting the double-layered roof. In vesivirus, the roof of the arched VP1 dimer was formed through the interactions between two antiparallel β strands (a and b) and between antiparallel loops (c), exhibiting the triple-layered roof. In lagovirus and norovirus, the roof of the arched VP1 dimer had a large hole (red asterisk) and was formed only with the side wall interactions consisting of the residues extending from the P1 subdomain.

In SaV, two conserved motifs, PPG and GWS, in VP1 are known (1). These can be found in other caliciviruses whose atomic structures have been reported (Fig. 8). The PPG motif is preserved in the VP1s of sapovirus, lagovirus, vesivirus, and norovirus, while GWS is conserved in the VP1s of these viruses, except for norovirus. The PPG motif was located at the interdomain interface between the S-domain dimers in these four viruses (Fig. 9), showing that the PPG motif commonly stabilized the inner capsid shell of these caliciviruses. Additionally, as described above, the NTAs also worked to stabilize connections between S domains, extending differently to the adjacent S-domain dimer for each calicivirus (black arrows in Fig. 9). Alternatively, the GWS motif is located at the boundary between the P1 and P2 subdomains in sapovirus, lagovirus, and vesivirus. Interestingly, these three caliciviruses commonly form an arched protruding dimer, where two P domains primarily interact through an external P2 subdomain. The stability of individual P domains is important for such arched conformations, and thus, the GWS motif is proposed to support this function. Notably, the loop between βB′ and α1′ in the P1 subdomain was extended to the external P2 subdomain in SaV (blue in the sapovirus panel in Fig. 9), directly interacting with the adjacent P2 subdomain from both sides of the P-domain dimer (asterisk in the sapovirus panel in Fig. 9). The βB′-α1′ loop was located on the opposite side of the GWS motif in the P domain and can stabilize the external P2 subdomain from both sides of the P-domain dimer with the GWS motif (right of the sapovirus panel in Fig. 9). This extending loop from the P1 subdomain is also found in four other caliciviruses (magenta in Fig. 8 and blue loops in Fig. 9), which is proposed to commonly play a stabilization function in the arched P-domain dimer.

SaV shows that the arched P-domain dimer of VP1 consisted of double layers of connections with the antiparallel loops and β strands (a and b in the sapovirus panel in Fig. 9), forming a thin roof (Fig. 5). Vesivirus exhibits a similar arched P-domain dimer of VP1, but it consists of triple layers of connections with one antiparallel loop and two antiparallel β strands, forming a thick roof (a to c in the vesivirus panel in Fig. 9). In lagovirus and norovirus, these antiparallel loops and β strands in the P2 subdomain are not closely aligned (red asterisks in the lagovirus and norovirus panels in Fig. 9). The two P2 subdomains are connected only at both sides with the loop in the P2 subdomain and the extended residues from the P1 subdomain (blue in the lagovirus and norovirus panels in Fig. 9), forming a large hole in the middle of the roof (red asterisks in the lagovirus and norovirus panels in Fig. 9).

**FIG 10:**
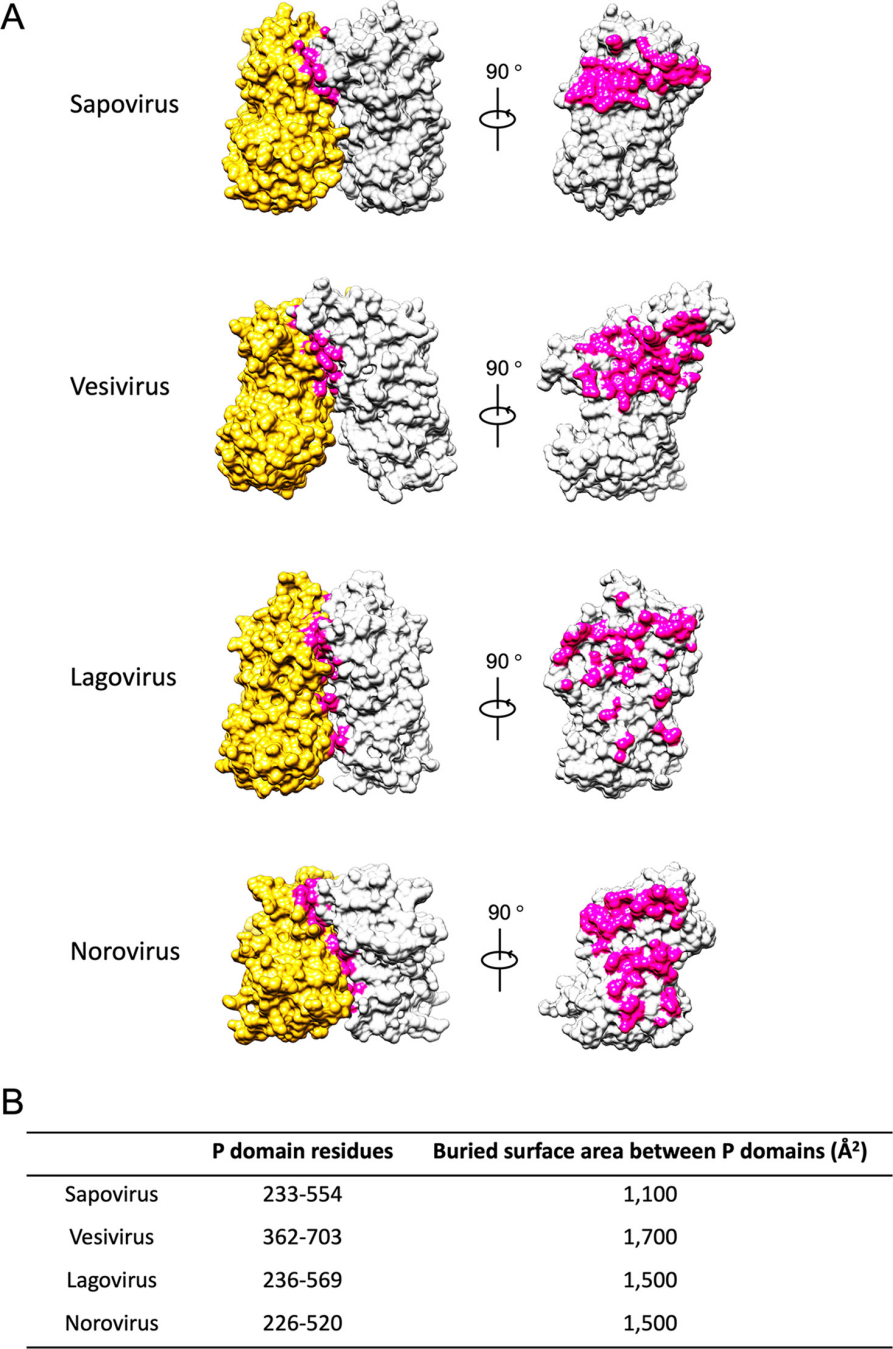
Buried surface of the VP1 dimer in four caliciviruses whose atomic structures have been reported. (A) Molecular surfaces of the VP1 dimers of sapovirus GI.6 (PDB accession number 7DOD), vesivirus (PDB accession number 2GH8), lagovirus (PDB accession number 3J1P), and norovirus (PDB accession number 1IHM). Buried surfaces of the P domain between the VP1 dimers are shown in magenta. (B) Buried surface areas of the P domains between the VP1 dimers were calculated and are listed with the numbers of P-domain residues in the four caliciviruses.

For further investigations, the buried surface caused by the intermolecular interaction between the two P domains was estimated in the SaV VP1 P-domain dimer and compared with those of other caliciviruses (Fig. 10). The buried surface was limited to the P2 subdomain in sapovirus and vesivirus. It was further restricted to a part of the P2 subdomain in sapovirus, while it was more dispersed in vesivirus. In lagovirus, the buried surface that primarily occupied in the P2 subdomain was partially expanded to the P1 subdomain, whereas it was dispersed across all interfaces of the P domain in norovirus. The calculated buried surface area between the P domains of SaV was 1.1 × 10^3^ Å^2^, which was significantly smaller than those of other caliciviruses (1.5 × 10^3^ to 1.7 × 10^3^ Å^2^) (Fig. 10B). In vesivirus, the largest buried area was identified to be 1.7 × 10^3^ Å^2^, although it was formed only on the P2 subdomain. These results suggest that SaV has a unique arched dimeric protrusion in calicivirus capsids. It is also expected that the use of SaV P-domain dimers as a vaccine antigen will require an additional stabilizing mechanism to maintain an intact P-domain dimer structure.

Recently, dynamic structural changes in capsids involved in viral infection have been reported for some caliciviruses. In murine norovirus, a dynamic rotation of the P domain has been reported to occur in response to aqueous conditions, including bile acids or metal ions, resulting in altered infectivity (11, 26). In feline calicivirus, the minor capsid protein of VP2 has been reported to form a portal-like assembly on the capsid surface after receptor engagement, suggesting that it functions as a channel for the delivery of the viral genome (16). However, such dynamic structural changes have not yet been observed in SaV. The recently established SaV culture systems using human cell lines require bile acids as a supplement (8). Although the function of the VP2 minor structure protein has not been elucidated in SaV, it has been suggested that in norovirus, VP2 can stabilize the secondary structure of VLPs under alkaline pH conditions (27). It may be necessary to investigate the structure of virions under various aqueous conditions, including bile acids or metal ions, and the structure of VLPs by coexpressing the VP1 and VP2 proteins in the future.

In this study, we successfully generated a stable VLP of HuSaV GI.6, making it possible for the first time to build an atomic model of the VP1 protein. So far, the VLP of the GI.5 and GI.1 chimeric strain was the most stable VLP acquired, revealing a subnanometer (8.5-Å)-resolution cryo-EM map (Electron Microscopy Data Bank [EMDB] accession number EMD-3281). The map was similar to the current 2.9-Å-resolution cryo-EM map compared at the same 8.5-Å resolution. The homology model built based on the vesivirus Protein Data Bank (PDB) model (PDB accession number 2GH8) showed similar domain and subdomain boundaries for the P1, P2, and S (sub)domains (18). This indicated that the current atomic model of HuSaV GI.6 represents a capsid structure common to HuSaVs. Therefore, the atomic model of the HuSaV GI.6 VLP can be widely used to construct its effective antigens and design antiviral drugs for the medical treatment of sapovirus infections.

## MATERIALS AND METHODS

### Expression of the HuSaV GI.6 VP1 protein in insect cells and purification of VLPs.

A baculovirus expression system constructed in a previous study (28) was employed in this study. To generate VLPs of the SaV GI strain, the recombinant baculovirus of HuSaV VP1 of the Nichinan strain was propagated in Sf9 cells (Thermo Fisher Scientific, USA) as described previously (28). The recombinant baculovirus was used to infect approximately 3 × 10^6^ confluent High Five cells (Thermo Fisher Scientific, USA) at a multiplicity of infection (MOI) of 5 to 10 in 1.5 mL Express Five serum-free medium (SFM) (Gibco, USA), and the infected cells were incubated at 26°C. The culture medium was harvested at 5 to 6 days postinfection (dpi), centrifuged for 10 min at 3,000 × *g*, and further centrifuged for 30 min at 10,000 × *g*. Finally, the VLPs were concentrated by ultracentrifugation for 2 h at 31,000 rpm at 4°C (Beckman SW-31Ti rotor) and then resuspended in 500 μL of Express Five SFM. Samples were examined for VLP formation by conventional electron microscopy after purifying VLPs through CsCl, as described previously (29).

### Cryo-EM data collection and processing.

**FIG 11:**
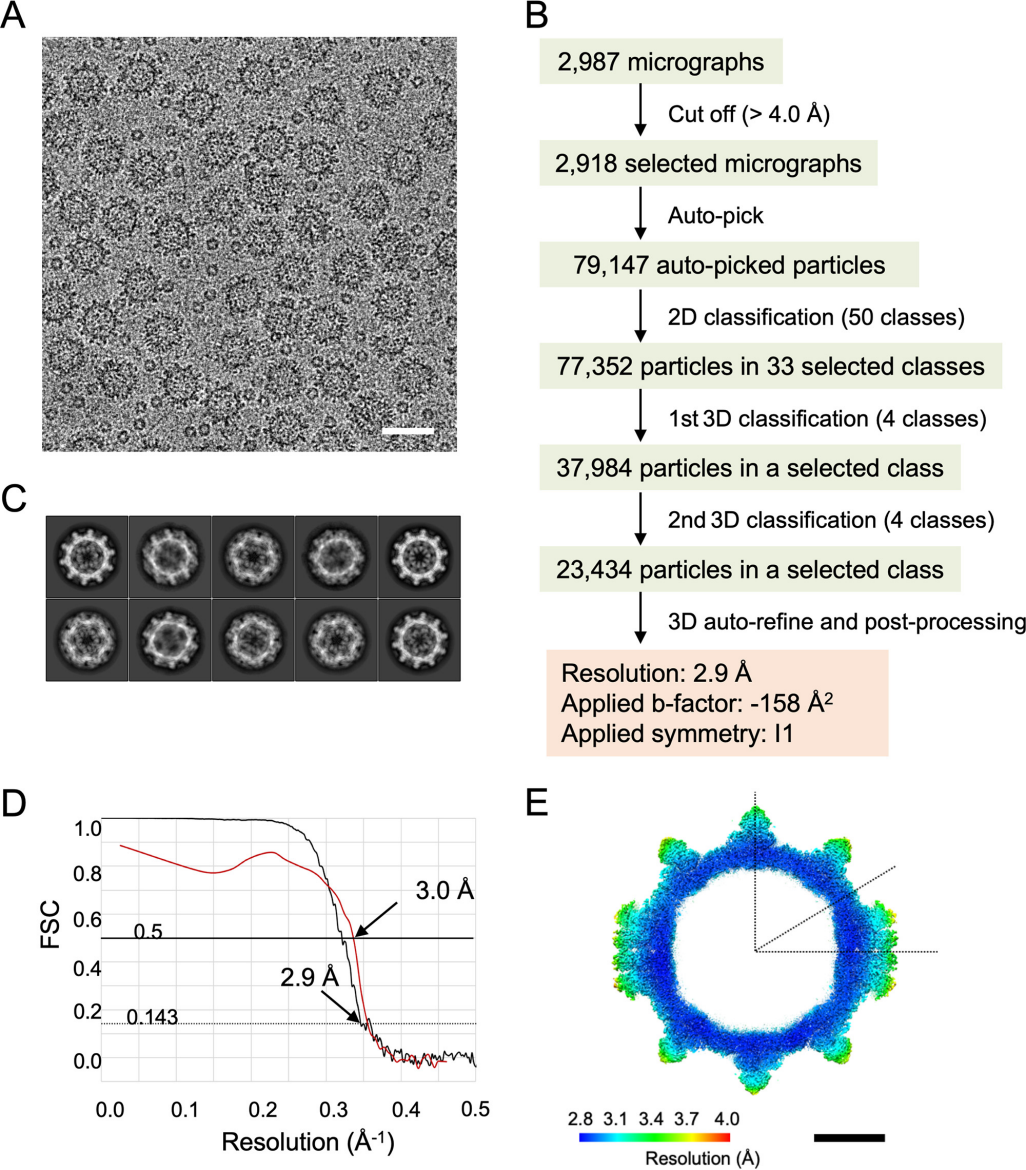
Cryo-EM data collection and processing. (A) Representative cryo-EM micrograph used for image analysis of the HuSaV GI.6 VLP. Bar, 50 nm. (B) Flowchart showing the cryo-EM structure determination of the HuSaV GI.6 VLPs. (C) Representative 2D class-averaged images for HuSaV GI.6 VLPs. (D) Gold-standard FSC curve calculated between independently refined half-maps of the reconstruction (black) and map-to-model FSC curve calculated between the cryo-EM map and the map generated from the atomic model (red). (E) Local resolution map of the HuSaV GI.6 VLP viewed at the central cross-section. Resolutions are indicated in the color map. Bar, 10 nm.

For cryo-EM experiments, 3 μL of a sample solution suspended in Express Five SFM was applied on a Quantifoil holey carbon grid (R1.2/1.3, Mo 200-mesh; Quantifoil Micro Tools GmbH, Germany) at 4°C and with 100% humidity and then plunge-frozen into liquid ethane using a Vitrobot Mark IV system (Thermo Fisher Scientific, USA). The cryo-EM grids were examined at liquid nitrogen temperature using a cryo-electron microscope (Titan Krios G2; Thermo Fisher Scientific, USA), incorporating a field emission gun and a Cs corrector (Corrected Electron Optical Systems GmbH, Germany). The microscope was operated at 300 kV and a nominal magnification of ×75,000. Movie frames were recorded using a Falcon II direct electron detector (Thermo Fisher Scientific, USA), applied with a nominal underfocus value ranging from −1.0 to −2.5 μm. An accumulated dose of 20 electrons per Å^2^ on the sample was fractionated into a movie stack of 16 image frames at 0.0625 s per frame, for a 1.0-s total exposure time. The workflow for cryo-EM image processing is summarized in Fig. 11B. Movies (0.87 Å/pixel) were subsequently aligned and summed using MotionCorr software (30) to obtain a final motion-corrected image (Fig. 11A). The contrast transfer function was estimated using the CTFFIND4 program (31). Micrographs exhibiting poor power spectra (based on the extent and regularity of the Thon rings) were rejected (4.0-Å-resolution cutoff). Approximately 2,000 particles were manually picked using EMAN2 (32) to generate two-dimensional (2D) classes as the templates for autopicking in Gautomatch (https://www2.mrc-lmb.cam.ac.uk/research/locally-developed-software/zhang-software/). All the following processes were conducted using RELION 2.1 software (33). A total of 79,147 autopicked particles from 2,918 micrographs were subjected to reference-free 2D classification. A total of 77,352 particles were selected from acceptable 2D classes (Fig. 11C) and subjected to two rounds of 3D classification with icosahedral symmetry. Finally, the 3D structure was reconstructed from 23,434 particles at a 2.9-Å resolution, estimated using the gold-standard Fourier shell correlation (FSC) with a 0.143 cutoff (34) (Fig. 11D). Local resolution variations were also calculated using RELION software (Fig. 11E). Data collection and processing statistics are shown in Table 2.

**TABLE 2:** Data collection and processing statistics^*a*^

Parameter	Value for sapovirus GI.6 VLP
Specimen data collection and processing statistics	
Microscope	FEI Titan Krios G2
Detector	Falcon II direct electron detector
Nominal magnification	×75,000
Voltage (kV)	300
Nominal defocus range (μm)	−1.00 to −2.50
Pixel size (Å)	0.870
Total electron dose (e^−^/Å^2^)	20
Exposure time (s)	1.0
No. of frames per image	16
No. of micrographs	2,987
No. of micrographs used for analysis	2,918
Initial no. of particles	79,147
No. of particles used for 3D reconstruction	23,434
Map resolution (Å)	2.9
Applied b-factor (Å^2^)	−158

Model building and refinement statistics	
Refined resolution (Å)	2.9
Map Cross-correlation (around atoms)	0.795
RMSD bond length (Å)	0.01
RMSD bond angles (°)	1.00
Ramachandran plot preferred regions (%)	5.64
Ramachandran plot allowed regions (%)	94.36
Ramachandran plot outlier regions (%)	0.0
Rotamer outliers (%)	0.15
Clashscore	6.68
MolProbity score	1.75

a RMSD, root mean square deviation.

### Atomic model building and three-dimensional homology mapping.

The 2.9-Å map was used for *de novo* atomic model construction of the VP1 protein in O (35). The initial atomic model was refined with the phenix.real_space_refine function in PHENIX (36) and manual adjustment in COOT (37). The final model was further validated using MolProbity (38). SaV VP1 protein sequences were aligned using CLUSTAL-W (39). Identical and similar amino acid residues were defined according to the Risler matrix (40) and were mapped onto the surface of the SaV VP1 protein from the GI Nichinan strain using UCSF Chimera and ChimeraX software (41, 42).

### Data availability.

The cryo-EM map of the HuSaV VLP of the GI.6 strain has been deposited in the Electron Microscopy Data Bank under accession number EMD-30793. Atomic coordinates for the atomic model of the VLP have been deposited in the Protein Data Bank under accession number 7DOD.
